# Sexual Harassment and Abuse in Young Adulthood and Mental Health Outcomes

**DOI:** 10.1001/jamanetworkopen.2026.32514

**Published:** 2026-09-08

**Authors:** Samuel J. Mann, Anthony Rodriguez, Eric R. Pedersen, Wendy M. Troxel, Daniel Siconolfi, Elizabeth J. D’Amico

**Affiliations:** 1 RAND, Arlington, Virginia; 2 RAND, Boston, Massachusetts; 3 Department of Psychiatry and Behavioral Sciences, Keck School of Medicine, University of Southern California, Los Angeles; 4 RAND, Pittsburgh, Pennsylvania; 5 RAND, Santa Monica, California

## Abstract

This cohort study analyzes mental health trajectories before and after experiences of sexual harassment and abuse among young adults.

## Introduction

Sexual harassment (nonphysical, eg, rumors or comments) and abuse (intimidation or physical contact) is common and associated with poorer mental health outcomes.^1,2,3^ Existing studies rely on retrospective or cross-sectional data, limiting assessment of how mental health changes dynamically around the timing of harassment or abuse.^1^ Characterizing mental health trajectories before and after sexual harassment and abuse is important for distinguishing consequences of sexual harassment and abuse from preexisting vulnerability.

## Methods

Data for this cohort study come from annual waves 8 through 17 of a longitudinal cohort followed annually from adolescence into young adulthood (eMethods in Supplement 1).^4^ Data were collected from 2015 to 2025 when respondents were aged 18 (wave 11) to 28 (wave 17) years, on average. Participants annually reported whether they had been exposed to 6 types of sexual harassment and abuse experiences in the past year (unwelcome sexual comments, sexual rumors, unwelcome touching, unwanted sexual pictures, sexual intimidation, and forced sexual activity). Past-year sexual harassment and abuse was defined as self-report of any of these experiences. Clinically significant depression and anxiety were defined as scores of 10 or greater on the Generalized Anxiety Disorder-7 and Patient Health Questionnaire-8, respectively.^5,6^

We estimated event-study linear probability models for each sexual harassment and abuse type, comparing participants who reported sexual harassment and abuse with participants who never reported sexual harassment and abuse across waves. Event time was defined relative to first report and collapsed at end points (±3 points), with the year immediately before first report as the reference period. Individual fixed effects accounted for stable individual characteristics, and wave fixed effects accounted for secular trends. Standard errors were clustered at the participant level. Statistical significance was defined as a 2-sided *P* < .05. Analyses were conducted in Stata/MP 18 (StataCorp). All participants provided written informed consent; study procedures were approved by the RAND Human Subjects Protection Committee. We followed the STROBE reporting guidelines.

## Results

The sample included 2108 individuals, with 456 reporting never having experienced sexual harassment and abuse and 2404 reporting having experienced sexual harassment and abuse. At baseline, the mean (SD) age of participants was 18.3 (0.8) years. Of all participants, 1416 (58.9%) were female, 1325 (46.3%) were Hispanic, 553 (19.3%) were non-Hispanic Asian, and 579 (20.2%) were non-Hispanic White. Women, sexual minorities, and those who had clinically significant depression or anxiety at baseline were more likely to ever report sexual harassment and abuse (Table).

**Table.** Baseline Sample Descriptives by Experience of Sexual Harassment and Abuse^a^

Characteristic	Respondents, No. (%) (N = 2108)		Statistical test^b^	
	Never experienced sexual harassment and abuse (n = 456 [2754 person-waves])	Ever experienced sexual harassment and abuse (n = 2404 [19 893 person-waves])	χ^2^ (*df*)	*P* value
Age, mean (SD), y	18.4 (0.8)	18.3 (0.8)	*t* = 0.60	.55
Race and ethnicity				
Hispanic	225 (49.3)	1,100 (45.8)	5.21 (4)	.27
Non-Hispanic Asian	94 (20.6)	459 (19.1)		
Non-Hispanic Black	7 (1.5)	57 (2.4)		
Non-Hispanic White	79 (17.3)	500 (20.8)		
Non-Hispanic other^c^	51 (11.2)	288 (12.0)		
Sex				
Female	144 (31.6)	1416 (58.9)	115.62 (1)	<.001
Male	312 (68.4)	987 (41.1)		
Educational attainment				
Less than high school	268 (58.8)	1804 (75.0)	21.16 (3)	<.001
High school or GED	99 (21.7)	505 (21.0)		
College degree	17 (3.7)	37 (1.5)		
Graduate degree	2 (0.4)	3 (0.1)		
Sexual orientation				
Heterosexual	428 (93.9)	2092 (87.0)	25.30 (3)	<.001
Gay or lesbian	10 (2.2)	70 (2.9)		
Bisexual	9 (2.0)	158 (6.6)		
Other	4 (0.9)	84 (3.5)		
Baseline clinically significant anxiety (GAD-7 ≥10)	17 (3.7)	301 (12.5)	20.74 (1)	<.001
Baseline clinically significant depression (PHQ-8 ≥10)	15 (3.3)	339 (14.1)	30.18 (1)	<.001

Abbreviations: GAD, Generalized Anxiety Disorder-7; GED, general educational development; PHQ, Patient Health Questionnaire-8.

^a^ Data come from 2806 respondents in wave 8 of the Study of Racial Diversity in Alcohol and Other Drug Use During the Transition to Adulthood cohort.^4^

^b^ χ^2^ Tests were used for categorical variables, and the Welch *t* test was used for continuous variables.

^c^ Race and ethnicity were self-reported. Non-Hispanic other includes American Indian, Native Hawaiian, and multiple races or ethnicities. Race and ethnicity were included to characterize the underlying sample.

The Figure shows little evidence of differential pretrends across sexual harassment and abuse types. First reports of unwelcome sexual comments were associated with a 3.4 (95% CI, 1.1-5.7) percentage point (pp; 22.8% relative to baseline) increase in clinically significant anxiety and a 3.2 (95% CI, 95% CI, 0.9-5.5) pp (19.0% relative to baseline) increase in clinically significant depression in the sexual harassment and abuse year. First reports of sexual rumors were associated with increases in anxiety (5.2 [95% CI, 2.0-8.3] pp; 34.9% increase) and depression (7.1 [95% CI, 3.8-10.4] pp; 42.3% increase) in the sexual harassment and abuse year, with anxiety increases persisting for 2 more years. Unwelcome touching was associated with increases in anxiety (5.1 [95% CI, 2.6-7.7] pp; 34.2% increase) and depression (3.8 [95% CI, 1.1-6.5] pp; 22.6% increase) in the sexual harassment and abuse year, with anxiety remaining elevated an additional year. Unwanted sexual pictures were associated with increased anxiety 1 year after sexual harassment and abuse (4.0 [95% CI, 0.8-7.2] pp; 26.8% increase); coefficients for depression were nonsignificant. Sexual intimidation showed the largest increases in anxiety (8.5 [95% CI, 5.0-12.0] pp; 57.0% increase) and depression (7.9 [95% CI, 4.2-11.5] pp; 47% increase), with effects persisting for at least 2 years after sexual harassment and abuse. Forced sexual activity was associated with increases in anxiety (5.9 [95% CI, 2.2-9.6] pp; 39.6% increase) and depression (6.0 [95% CI, 2.0-9.9] pp; 35.7% increase), with anxiety remaining elevated 1 year later.

**Figure.**
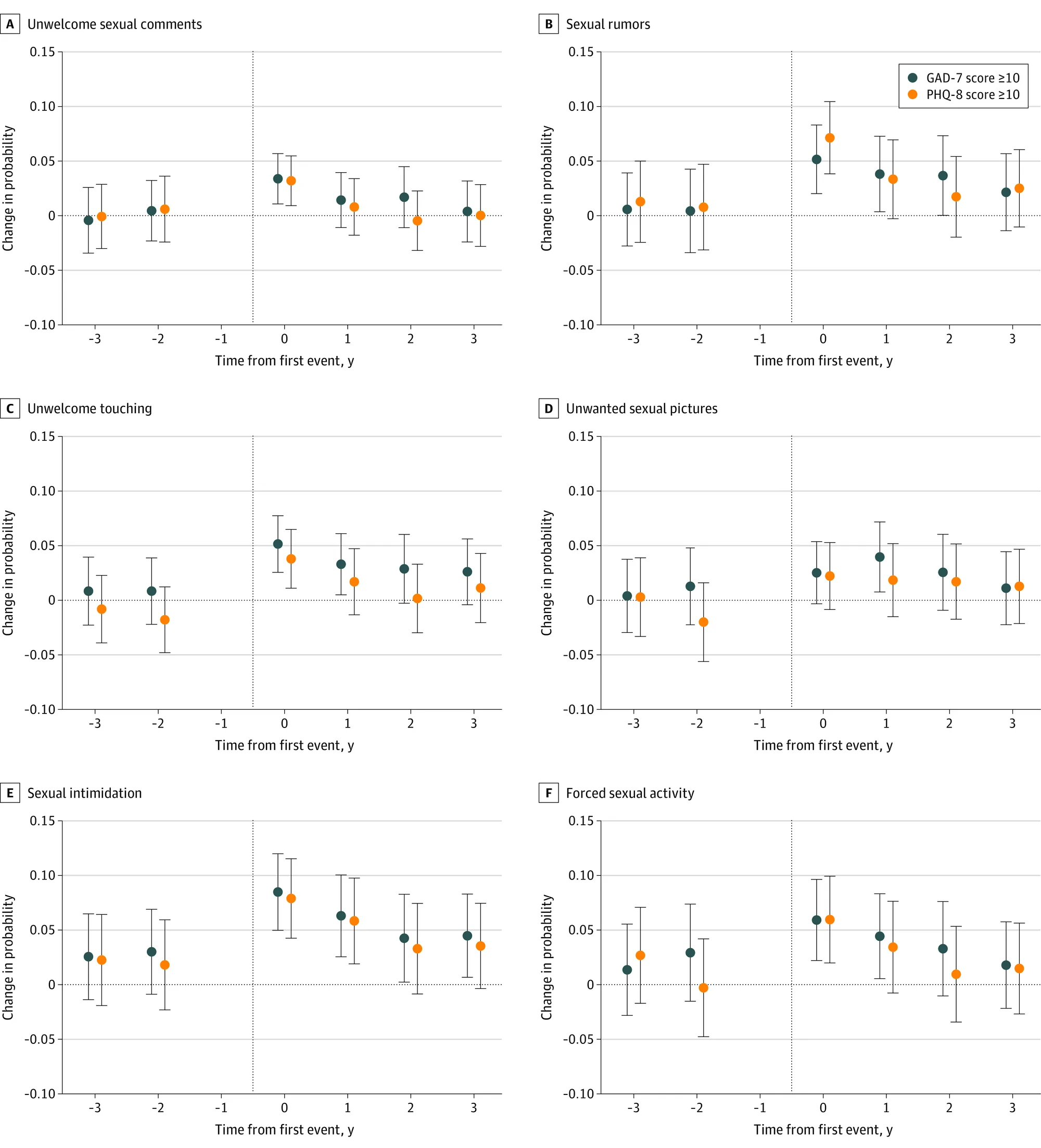
Sexual Harassment and Abuse and Mental Health Event Studies Estimates are from separate event-study linear probability models for each sexual harassment and abuse type. Outcomes were clinically significant anxiety (blue dots; Generalized Anxiety Disorder-7 [GAD-7] score ≥10) and clinically significant depression (orange dots; Patient Health Questionnaire-8 [PHQ-8] score ≥10). Each model compared participants who reported sexual harassment and abuse with participants who never reported any sexual sexual harassment and abuse across waves 8 through 17. Event time was defined relative to the first year the sexual harassment and abuse type was reported and collapsed at 3 or more years before the sexual harassment and abuse and 3 or more years after the sexual harassment and abuse. The omitted reference period was the year immediately before first sexual harassment and abuse. Models included participant and wave fixed effects. Participant fixed effects accounted for fixed individual characteristics such as sex and race. Standard errors were clustered at the participant level. Error bars indicate 95% CIs.

## Discussion

In this cohort study of young adults, sexual harassment and abuse was associated with increases in clinically significant depression and anxiety. Associations emerged around the timing of sexual harassment and abuse and persisted for most sexual harassment and abuse types. Associations were most persistent for anxiety and largest in magnitude for sexual intimidation, suggesting that sexual harassment and abuse may adversely affect mental health even when it does not involve forced sexual activity.

This study extends prior work that has lacked pre–sexual harassment and abuse mental health assessment or controls who did not report sexual harassment and abuse.^1^ Findings have implications for early identification and support. Given the high incidence of sexual harassment and abuse among young adults,^3^ clinicians should identify affected individuals soon after experiencing sexual harassment and abuse and ensure timely intervention, particularly because anxiety and depression may persist over time.

Results suggest that adverse mental health consequences are not limited to physical sexual assault (ie, rape) but extend to other forms of sexual harassment and abuse. Clinical guidance is often framed around sexual assault,^7^ whereas evidence suggests that broader assessment approaches may improve identification of other sexual harassment and abuse experiences that might be missed.^8^ Comprehensive, trauma-informed assessment and response strategies across clinical, educational, and other young adult–serving settings may improve identification, reduce retraumatization, and expand access to timely support and intervention.^9,10^

Despite strengths of this study, there are limitations. Sexual harassment and abuse were self-reported, which may introduce recall error. Forced sexual activity was not further disaggregated (eg, into forcible rape or alcohol- or drug-facilitated sexual assault). Outcomes were based on screening thresholds rather than clinical diagnoses. Additionally, participants may have experienced subsequent sexual harassment and abuse after their first reported exposure, and postevent estimates may reflect the consequences of repeated or ongoing sexual harassment and abuse rather than a single isolated event.

## Notes

Supplement 1. eMethods. eReferences.

Supplement 2. Data Sharing Statement
