# Supplementary material for: Sexual Harassment and Abuse in Young Adulthood and Mental Health Outcomes
Source: JAMA Netw Open. 2026 Sep 8;9(9):e2632514. doi: 10.1001/jamanetworkopen.2026.32514 (PMC13555369; doi:10.1001/jamanetworkopen.2026.32514)
Supplement: Supplementary file 1 [file jamanetwopen-e2632514-s001.pdf]

Supplemental Online Content

Mann SJ, Rodriguez A, Pederson ER, Troxel WM, Siconolfi D, D'AMico EJ. Sexual harassment and abuse in young adulthood and mental health outcomes. *JAMA Netw. Open*. 2026;9(9):e2632514. doi:10.1001/jamanetworkopen.2026.32514

eMethods.

eReferences.

This supplemental material has been provided by the authors to give readers additional information about their work.

© 2026 Mann SJ et al. *JAMA Network Open*.

eMethods.

Study design and cohort

Data came from the STRATA cohort, an ongoing longitudinal study. Participants were originally recruited in 2008 (average age = 11.5) from 16 Southern California middle schools as part of CHOICE, a voluntary after-school alcohol and other drug prevention trial. After the first year, intervention effects dissipated and are not expected to influence current outcomes.[1] Individuals were representative of the broader student bodies within Southern California middle schools in terms of demographic characteristics and future substance use risk. Across waves 1 through 5, participants completed surveys during middle school classes. After they transitioned from middle school to high school, following wave 5, participants were re-contacted and re-consented to complete annual web-based surveys. At wave 6, 61% of the wave 5 sample participated in the follow-up survey (n=2,653).

For the present study, we used data from annual waves 8 through 17, the study period in which both sexual harassment/abuse and mental health measures used in this study were available in a consistent format. Surveys were administered online, and participants were compensated for survey completion. Surveys were conducted yearly. Average wave to wave retention across waves 8 to 17 was 90.79%. All participants consented to the study, and procedures were approved by the RAND Human Subjects Protections Committee.

Across all waves (8-17), respondents were asked "During the past year, how did someone else do the following things to you?: (1) made unwelcome sexual comments, jokes, or gestures, (2) spread sexual rumors about you, (3) touched you in an unwelcome way, (4) showed you sexy or sexual pictures that you didn't want to see, (5) physically intimidated you in a sexual way, (6) forced you to do something sexual when you did not want to. Respondents that responded with any number greater than zero were defined as experiencing sexual harassment/abuse in the prior year.

Analytic sample and event-time construction

The analytic sample was constructed in person-wave format. For each sexual harassment/abuse–specific analysis, the analytic sample included: (1) participants who reported first exposure to that sexual harassment/abuse type during the study period and (2) a comparison group restricted

© 2026 Mann SJ et al. *JAMA Network Open*.

to participants who never reported any sexual harassment/abuse of any type during the study period. For each sexual harassment/abuse type, event time was defined relative to the first year of reported past-year exposure. To improve precision and reduce sparsity in the tails of the distribution, event time was collapsed at the endpoints: 3 or more years before first exposure was coded as −3, and 3 or more years after exposure was coded as 3. The year immediately before first exposure served as the omitted reference category in all event-study models. All event-study models were estimated using all available person-wave observations within each analytic sample, without restricting to a balanced panel, to improve sample size and precision. Individuals contributed observations for as many waves as they participated: mean = 9 of the available 10 waves.

Sexual harassment/abuse types were not mutually exclusive. Participants who reported more than one type of sexual harassment/abuse could contribute to multiple sexual harassment/abuse–type-specific analyses (see below). Accordingly, estimates for a given sexual harassment/abuse type should be interpreted as changes associated with first reported exposure to that type, rather than of an isolated or mutually exclusive sexual harassment/abuse experience.

Statistical analysis

We estimated separate event-study linear probability models for each sexual harassment/abuse type and each outcome. We estimated the following model separately for each sexual harassment/abuse type and each mental health outcome:

$$Y_{it} = \sum_{k \neq -1} \beta_k \, D_{it}^{k} + \alpha_i + \lambda_t + \epsilon_{it}$$

where Y_{it} is an indicator for moderate anxiety or clinically significant depression, for participant i in survey wave t. D_{it}^{k} is an indicator for event time k relative to first reported sexual harassment/abuse, α_i represents participant fixed effects, and λ_t represents survey-wave fixed effects. Participant fixed effects were implemented as participant-specific indicators and therefore absorbed all characteristics that were stable within individuals over time, including sex, race/ethnicity, baseline family background, and persistent differences in mental health vulnerability, while wave fixed effects were implemented as wave-specific indicators and account for temporal trends and period shocks common to all participants.

© 2026 Mann SJ et al. *JAMA Network Open*.

Event time was collapsed at endpoints, with $k = -3$ representing 3 or more years before first reported sexual harassment/abuse and $k = 3$ representing 3 or more years after first reported sexual harassment/abuse. The year immediately before first reported sexual harassment/abuse ($k = -1$) was omitted as the reference period. For participants who never reported any sexual sexual harassment/abuse during waves 8 through 17, all event-time indicators were coded as 0 in all waves.

This approach also allows us to visually and statistically assess whether mental health outcomes were already changing before first reported sexual harassment/abuse, and trace how outcomes changed after sexual harassment/abuse. Standard errors were clustered at the participant level to account for repeated observations within individuals across waves.

© 2026 Mann SJ et al. *JAMA Network Open*.

eReferences

1. D'Amico EJ, Tucker JS, Miles JNV, Zhou AJ, Shih RA, Green HD. Preventing alcohol use with a voluntary after-school program for middle school students: results from a cluster randomized controlled trial of CHOICE. *Prev Sci*. 2012;13(4):415-425. doi:10.1007/s11121-011-0269-7

© 2026 Mann SJ et al. *JAMA Network Open*.
