# Supplementary material for: Sexual Harassment and Abuse in Young Adulthood and Mental Health Outcomes
Source: JAMA Netw Open. 2026 Sep 8;9(9):e2632514. doi: 10.1001/jamanetworkopen.2026.32514 (PMC13555369; doi:10.1001/jamanetworkopen.2026.32514)
Supplement: Supplementary file 2 [file jamanetwopen-e2632514-s002.pdf]

# Data Sharing Statement

Mann. Sexual Harassment and Abuse in Young Adulthood and Mental Health Outcomes.
*JAMA Netw Open*. Published September 08, 2026. doi:10.1001/jamanetworkopen.2026.32514

**Data**
**Data available:** No

**Additional Information**
**Explanation for why data not available:** Study is ongoing
